# Morphological, Molecular Identification and Pathogenicity of *Neoscytalidium dimidiatum* Causing Stem Canker of *Hylocereus polyrhizus* in Southern Thailand

**DOI:** 10.3390/plants11040504

**Published:** 2022-02-12

**Authors:** Kim Sreang Dy, Prisana Wonglom, Chaninun Pornsuriya, Anurag Sunpapao

**Affiliations:** 1 Agricultural Innovation and Management Division (Pest Management), Faculty of Natural Resources, Prince of Songkla University, Hatyai 90110, Songkhla, Thailand; dykimsreang168@gmail.com (K.S.D.); chaninun.p@psu.ac.th (C.P.); 2 Faculty of Technology and Community Development, Thaksin University, Pa Payom 93210, Phatthalung, Thailand; prisana.w@tsu.ac.th

**Keywords:** morphology, molecular identification, pathogenicity test, pitaya

## Abstract

Red-fleshed dragon fruit (*Hylocereus polyrhizus*) is commonly cultivated in Thailand, especially in southern Thailand, where the weather favors plant growth and development. In 2021, stem canker of *H. polyrhizus* was observed in a dragon fruit plantation field in Phatthalung Province, southern Thailand. Small, orange circular spots developed on the stem of *H. polyrhizus*, which later became gray, and the lesion expanded with a mass of conidia. *Scytalidium*-like fungus was isolated from infected tissues. Based on morphology and phylogenetic analyses of internal transcribed spacer (ITS), nuclear large subunit (LSU) and β-tubulin (*tub*) sequences of fungal isolates, the fungus was identified as *Neoscytalidium dimidiatum*. Pathogenicity tests revealed that this isolate caused stem canker on the stem of *H. polyrhizus,* similar to that observed in the field. Knowledge of the diagnosis of plant diseases is an important step for managing plant diseases and therefore, this finding provides basic information for the development of appropriate strategies to manage stem canker disease on *H. polyrhizus* plants.

## 1. Introduction

Dragon fruit (*Hylocereus* spp.) is native to Latin America’s tropical and subtropical forest regions, including North, Central, and South America. Dragon fruit, of the genus *Hylocereus*, belongs to the Cactaceae family of climbing cactus [1] and is known by several names, including pitaya, pitahaya, and strawberry pear. Dragon fruit enriched with micronutrients is in high demand and is being promoted as a healthy fruit [2]. Currently, dragon fruit is classified into one of three varieties: *H. polyrhizus*; *H. undatus*; or *Selenicereus megalanthus* [3]. The suitability of a tropical climate, rainfall requirements, and soil types may have contributed to the growth of dragon fruit, especially red-fleshed dragon fruit [3].

Due to increased planting areas and high demand, dragon fruit in many crops attract disease and pests. Several diseases have been reported to negatively affect dragon fruit plantations and production [4]. Disease caused by fungi is a major problem for dragon fruit plantations worldwide [3,5]. For instance, the fungus *Colletotrichum gloeosporioides* was found to cause anthracnose on dragon fruit in Malaysia [3]. The fungi *Neoscytalidium dimidiatum* and *Bipolris* sp. have been reported to cause canker and bipolaris black spot, respectively, on dragon fruit in Vietnam [5]. Furthermore, the fungus *Gilbertella persicaria* was recently reported to cause flower rot on red-fleshed dragon fruit in Thailand [6].

Dragon fruit is an economically important crop that can support additive income for many households in Thailand. Saradhuldhat et al. [7] demonstrated that dragon fruit could grow in practically any type of soil, both upland and lowland in Thailand, and they are distributed throughout Thailand. Thailand is located in tropical and subtropical regions where the weather favors disease spread [8,9]. Several emerging diseases have been isolated and reported to cause diseases in several plant species in this area in the past 5 years [10,11,12,13,14,15,16,17]. However, the identification of fungal pathogens causing diseases on *H. polyrhizus* in Thailand is rarely documented. During 2021, stem canker of red-fleshed dragon fruit (*H. polyrhizus*) was observed in a cultivation field in Phatthalung Province, southern Thailand. Therefore, this research aimed to identify pathogens causing stem canker in Thailand based on morphology, molecular properties, and pathogenicity tests.

## 2. Results

### 2.1. Symptom Observation

**Figure 1:**
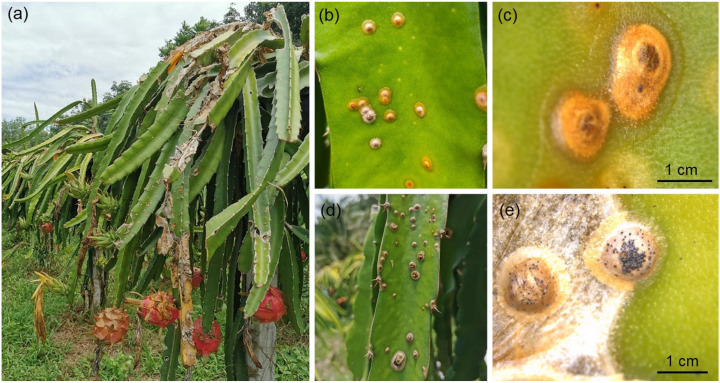
Stem canker of *Hylocereus polyrhizus* in the field (**a**), whitish-yellow spots, coalesced to form larger spots or lesions (**b**), spots or lesions surrounded by yellow halos (**c**), black pycnidia on stem of *H. polyrhizus* (**d**,**e**).

Sunken brown necrosis was observed on the stems of *H. polyrhizus* at the cultivation field in Phatthalung Province, southern Thailand (Figure 1a). The whitish-yellow spots turned orange to brown with age. The spots coalesced to form larger spots or lesions and were surrounded by yellow halos (Figure 1b,c). The spots turned into necrotic water-soaked lesions and produced black pycnidia on the stem of *H. polyrhizus* (Figure 1d,e). The fungus was directly isolated and cultured on PDA for further study.

### 2.2. Morphology of Fungal Isolate

**Figure 2:**
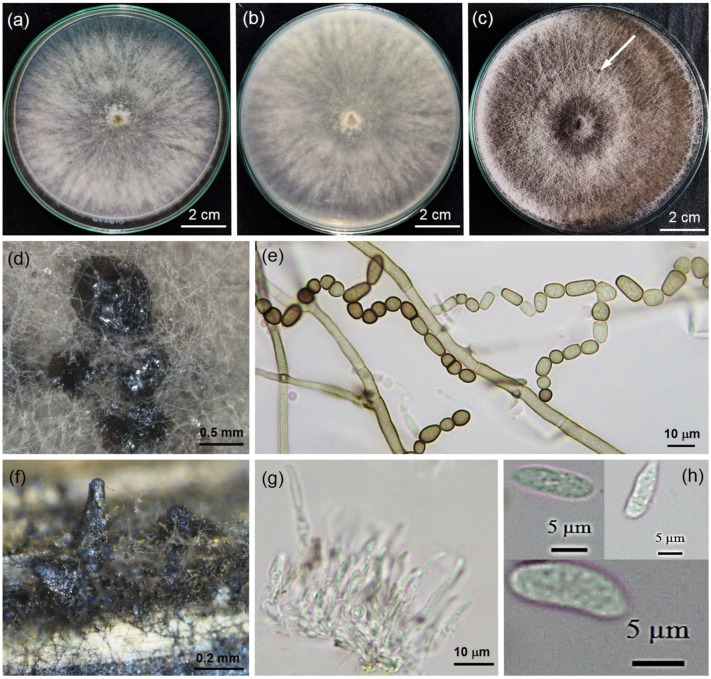
Morphological characteristics of PSU-SC02 on *Hylocereus polyrhizus*, 3-day-old colony on PDA in top (**a**) and bottom view (**b**), 4-week-old colony on PDA developed small black conidiomata ((**c**), arrow), zoom view of conidiomata (**d**), hyphae and arthroconidia (**e**), pycnidia developed on dried Napier grasses (**f**), conidiogenouse cells (**g**), and pycnidial conidia (**h**).

The fungal isolate PSU-SC02 obtained from PDA stock in Section 2.1 showed hairy colonies and olive-green to grayish colonies with dark gray to black pigmentation on PDA (Figure 2a–c). PSU-SC02 reached a diameter of 9 cm on PDA plates within 3 days, and the growth rate was 3 cm/day. The morphology of the PSU-SC02 isolate showed *Scytalidium*-like fungus. The hyphae were brown, branched, septate, and constricted into spore chains before disarticulation into arthroconidia. The arthroconidia were ellipsoid to ovoid in shape and hyaline to dark brown with thick walls and septate arthrospores, 3.1–18.0 μm long × 3.8–10.3 μm wide (*n* = 20, av = 10.0 ± 3.7 × 5.4 ± 1.8) μm (Figure 2e). Pycnidia had rarely developed on PDA after 4 weeks of incubation and developed on dried Napier grasses within 1 week (Figure 2d,f). Conidiogenous cells were observed in pycnidia that developed on dried Napier grass (Figure 2g). Pycnidial conidia were aseptate, ellipsoidal to nearly fusiform, and 8.5–15.5 μm long × 3.5–5.7 μm wide (*n* = 20, av = 12.5 ± 1.7 × 4.7 ± 0.6) μm (Figure 2h). The fungal isolate was deposited in the Culture Collection of Pest Management, Faculty of Natural Resources, Prince of Songkla University Thailand, with accession number PSU-SC02.

### 2.3. Molecular Identification

**Figure 3:**
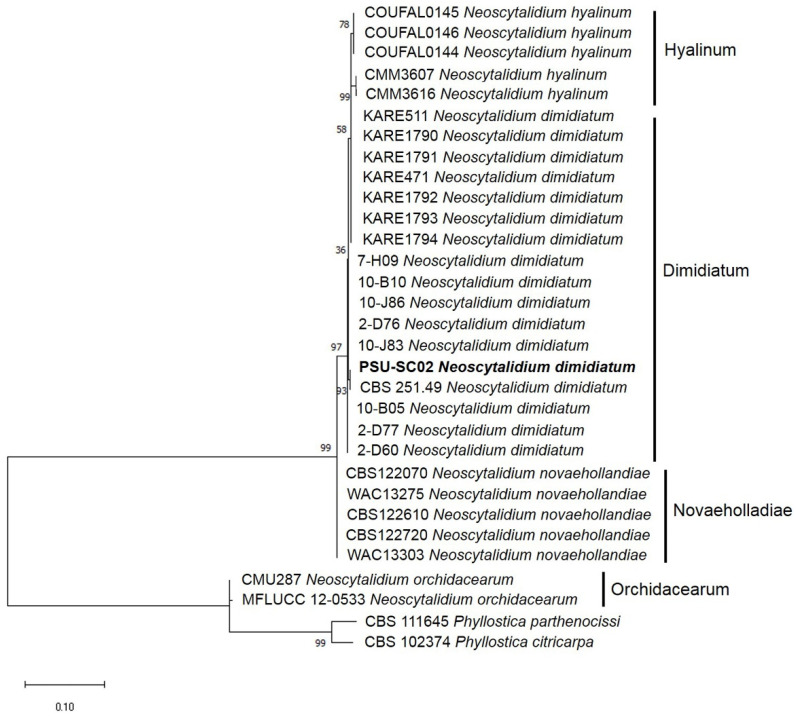
Phylogenetic tree of combined DNA sequences (ITS, LSU, and *tub*) of *Neoscytalidium dimidiatum* and related species acquired from Genbank constructed by maximum likelihood with 1000 bootstrap replications. Bold letters indicate the sample from this study. *Phyllostica parthenocissi* and *P. citricarpa* were used as out groups.

The PCR products of ITS, LSU, and *tub* were approximately 917, 1326, and 411 base pairs (bp) long, respectively. A BLAST search (https://blast.ncbi.nlm.nih.gov, accessed on 10 December 2021) revealed ITS, LSU, and *tub* sequences identical to those of *Neoscytalidium dimidiatum,* with 99.43%, 100%, and 99.76% identity, respectively. The DNA sequences of ITS, LSU, and *tub* of PSU-SC02 were deposited in GenBank and acquired accession numbers LC660640, LC660641, and LC660642, respectively. The maximum likelihood (ML) tree of the combined DNA sequences of ITS, LSU, and *tub* showed that the PSU-SC02 isolate grouped in the same clade as *N. dimidiatum* CBS 251.49 (Figure 3). Therefore, fungal isolate PSU-SC02 was identified as *N. dimidiatum*.

### 2.4. Neoscytalidium dimidiatum Causing Stem Canker

**Figure 4:**
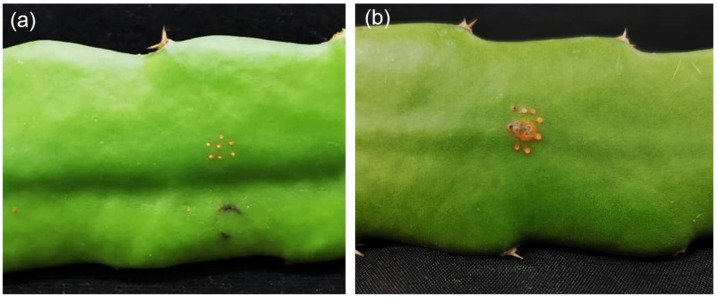
Pathogenicity test of *Neoscytalidium dimidiatum* PSU-SC02 on stem of *Hylocereus polyrhizus*, control (**a**), and PSU-SC02-inoculated stem (**b**).

To fulfill Koch’s postulates, a pathogenicity test was conducted on the stem of *H. polyrhizus*. Use of the agar plug method showed that *N. dimidiatum* PSU-SC02 caused cankers on healthy stems of *H. polyrhizus* after incubation in a moist box for 7 days (Figure 4). After 10 days of incubation, black pycnidia developed on the surface of *H. polyrhizus*, and the disease became severe when incubated for 14 days. Reisolation using the tissue transplanting method revealed that the morphology of the isolated fungus was similar to that of PSU-SC02.

## 3. Discussion

Canker disease caused by *Neoscytalidium* species is considered the most destructive disease for dragon fruit plantations worldwide and affects dragon fruit production, resulting in losses of up to 60–80% of market value [18]. In this study, we used both morphological and molecular tools to identify fungal pathogens at the species level and pathogenicity tests to fulfill Koch’s postulates. Based on morphology and phylogenetic analyses of ITS, LSU, and *tub* sequences in this study, the fungal pathogen causing stem canker on *H. polyrhizus* was identified as *N. dimidiatum*.

*Neoscytalidium dimidiatum* has been reported to be a fast-growing fungus that commonly reaches a diameter of 9 cm on Petri dishes within 3 days of incubation, as previously described by Mohd et al. [3] and Turkolmez et al. [19]. Our results are in agreement with previous research; the *N. dimidiatum* PSU-SC02 colony on PDA reached a diameter of 9 cm in 3 days. Arthroconidia of our strain (*N. dimidiatum* PSU-SC02) were diverse in size and shape, as previously reported for *N. dimidiatum* by Nouri et al. [20]. Pycnidia of *N. dimidiatum* have been observed on PDA with irregular shapes, singly or in aggregate after 4 weeks of incubation [20]; these characteristics were also observed in our study. Pycnidia was successfully developed on sterile dried grasses covered on WA (Figure 2f), as observed in previous studies [21]. Furthermore, *N. dimidiatum* PSU-SC02 from our study showed hyaline conidiogenous cells, and pycnidial conidia were aseptate, hyaline, and ellipsoidal to fusiform. These morphologies were typical of *N. dimidiatum*, as indicated by previous reports [3,19,22].

To confirm the *Neoscytalidium* species at the species level, multiple DNA sequences of ITS, LSU, and *tub* were analyzed in this study. It is known that the identification of fungal pathogens relies on both morphology and molecular properties. Mohd et al. [3] used the morphology and single DNA sequence of ITS to identify *N. dimidiatum* as causing stem canker on *H. polyrhizus* in Malaysia. Huang et al. [23] studied the morphology and phylogeny of double DNA sequences of ITS and LSU to identify a new species of *Neoscytalidium* and named it *N. orchidacearum*. Furthermore, Nouri et al. [20] also used the morphology and multiple DNA sequences of ITS, translation elongation factor 1-α (*tef1-α*), and *tub* to diagnose *N. dimidiatum* as causing canker, shoot blight, and fruit rot of almond in California. Based on our study, the morphology and molecular characteristics of ITS, LSU, and *tub* successfully identified *Scytalidium*-like fungi causing canker on *H. polyrhizus* as *N. scytalidium*.

Currently, fungi in the genus *Neoscytalidium* comprise four species, namely *N. dimidiatum*, *N. hyalinum*, *N. novaehollandiae,* and *N. orchidacearum*. *N. dimidatum* causes diseases in several plant species: canker of *Ficus* trees in Egypt [24]; root rot of sweet potato in Brazil [25]; shoot and needle blight of pines (*Pinus* spp.) in Turkey [26]; and shoot blight of citrus in Jordan [27]. Furthermore, *N. dimidiatum* has also been reported to cause canker on *H. polyrhizus* in Taiwan [28], Malaysia [3], and China [29]. However, there are no previous reports of *N. dimidiatum* causing canker on *H. polyrhizus* in Thailand. To our knowledge, this is the first report of *N. dimidiatum* as a fungal pathogen of canker on *H. polyrhizus* in Thailand.

## 4. Materials and Methods

### 4.1. Sample Collection and Pathogen Isolation

A total of ten symptomatic stem canker samples of *H. polyrhizus* were collected from a dragon fruit plantation field in Phatthalung Province, southern Thailand (7°45′24.2″ N, 99°58′47.2″ E), kept in a plastic bag in an ice box and taken to a plant pathology laboratory, where isolation was subsequently conducted. The isolation of fungal pathogens was conducted by tissue transplantation according to the method of Pornsuriya et al. [14]. Small pieces (2–3 mm) of infected tissue were surface disinfected with 0.5% sodium hypochlorite (NaOCl) [13], rinsed three times with sterilized distilled water (DW), air-dried, placed on water agar (WA), and incubated for 24 h at 28 ± 2 °C. Hyphal tips were cut and transferred to potato dextrose agar (PDA), incubated at ambient temperature (28 ± 2 °C) with natural light–light cycle and subsequently used for further methods.

### 4.2. Morphology Study

The morphology of fungal colonies is determined by their ability to grow on PDA, with varied observations of colony traits, such as color, size, and shape. In this study, three plates were incubated at 28 ± 2 °C, and the diameters of colonies were measured daily until the colonies reached the edge of the plate. The growth rate per day on PDA of fungal isolates was calculated. The growth rate experiment was repeated twice. The general morphological characteristics of the fungal isolates were observed using a Leica S8AP0 stereomicroscope (Leica Microsystems, Wetzlar, Germany) with 10× magnification and a Leica DM750 compound microscope (Leica Microsystems, Wetzlar, Germany) with 40× magnification. Mycelial plugs (0.5 cm) were cut from the edges of 3-day-old colonies of fungal isolates and placed on WA covered with sterile dried Napier grasses as substrates to develop pycnidia structures according to previous studies [13,21].

### 4.3. DNA Extraction and PCR Amplification

Fungal isolates were cultured on PDA for 2 days and subjected to DNA extraction by the mini-preparation method [30]. DNA quantification was observed by 1% agarose gel electrophoresis. PCR amplification of internal transcribed spacer (ITS), nuclear large subunit (LSU), and β-tubulin (*tub*) was amplified by using ITS1/ITS4 [31], LR0R/LR5 [32], and Bt2a/Bt2b [33] primer pairs, respectively. The PCR mixture was composed of 20 pmol of primers, DNA template, nuclease-free DW, and 2 × OneTaq^®^ 2X PCR master mix with standard buffer (Biloabs, New England, MA, USA). The Thermal Cycler (Bio-Rad Laboratories, CA, USA) was run with the following settings: initial denaturation at 94 °C for 30 s; 30 cycles of denaturation at 94 °C for 30 s, annealing at 60 °C for 60 s; extension at 68 °C for 1 min; and final extension at 68 °C for 5 min. PCR products were stained with novel juice (GeneDireX, Taoyuan, Taiwan), separated by 1% agarose gel electrophoresis and observed on an LED Transilluminator (GeneDireX, Taoyuan, Taiwan).

PCR products were sequenced at the WARD MEDIC sequencing service in Thailand. The DNA sequences of ITS, LSU, and *tub* were searched for in the Blast program (National Center for Biotechnology Information, NCBI). The DNA sequences were aligned by Clustal W, and a phylogenetic tree was constructed by MEGA X [34] with a maximum likelihood of 1000 bootstrap replications. DNA sequences of fungal isolates and related species were obtained from GenBank (Table 1) to construct the phylogenetic analyses. DNA sequences were deposited in GenBank to obtain accession numbers.

### 4.4. Pathogenicity Test

The fungal isolate was cultured on PDA for 7 days and subjected to inoculation of the stem of *H. polyrhizus* using the agar plug method [16]. Four stems of *H. polyrhizus* (4 replications) were prepared for inoculation, and the experiment was repeated twice. The stem of *H. polyrhizus* was disinfected with 70% ethanol and wounded with fine needles (0.5 cm in diameter). An agar plug of fungal isolate was cut from a 7-day-old colony and directly placed on wounded *H. polyrhizus* stems. Inoculation of PDA alone via agar plugs was used as a control. The inoculated samples were then incubated in a moist chamber to maintain humidity (85% RH), 28 ± 2 °C, with a natural light–dark cycle for 7 days. The development of canker was observed and photographed. Infected tissue samples were reisolated via the tissue transplanting method as described in Section 4.1, and morphology was observed via microscopy as explained in Section 4.2.

## 5. Conclusions

Herein, we identified the fungal pathogen causing stem canker in *H. polyrhizus* in Thailand. Based on the morphological characteristics and molecular properties of multiple DNA sequences of ITS, LSU, and *tub*, the pathogenicity tests revealed that the fungal pathogen *N. dimidiatum* caused stem canker in *H. polyrhizus*. Knowledge of the diagnosis of plant diseases is important for disease control and management. In order to determine and verify appropriate methods to manage stem canker disease on *H. polyrhizus* plants, further studies are needed in the near future.

## Figures and Tables

**Table 1:** DNA sequences used to generate a phylogenetic tree acquired from GenBank with accession numbers.

Taxa	Isolate	Host, Region	Accession Numbers		
			ITS	LSU	*tub*
*Neoscytalidium dimidiatum*	2-D60	*Ficus carica*, USA	MG021571	–	MG021514
	2-D76	*Prunus dulcis*, USA	MG021583	–	MG021480
	2-D77	*P. dulcis*, USA	MG021584	–	MG021481
	7-H09	*P. dulcis*, USA	MG021587	–	MG021484
	10-B05	*P. dulcis*, USA	MG021589	–	MG021486
	10-B10	*P. dulcis*, USA	MG021591	–	MG021488
	10-J83	*P. dulcis*, USA	MG021595	–	MG021492
	10-J86	*P. dulcis*, USA	MG021596	–	MG021493
	CBS 251.49	*Juglans regia*, USA	KF531819	DQ377923	FM211166
	KARE471	*P. dulcis*, USA	MG021601	–	MG021498
	KARE511	*P. dulcis*, USA	MG021608	–	MG021505
	KARE1790	*P. dulcis*, USA	MG021578	–	MF991145
	KARE1791	*P. dulcis*, USA	MG021579	–	MG021476
	KARE1792	*Prunus dulcis*, USA	MG021580	–	MG021477
	KARE1793	*P. dulcis*, USA	MG021581	–	MG021478
	KARE1794	*P. dulcis*, USA	MG021582	–	MG021479
	PSU-SC02 *	***Hylocereus polyrhizus***, **Thailand**	**LC660640**	LC660641	**LC660642**
*N. hyalinum*	CMM3607	*Jatropha curcas*, Brazil	KF234542	–	KF254925
	CMM3616	*J. curcas*, Brazil	JQ927342	–	KF254931
	COUFAL0144	*Nopalea cochenillifera*, Brazil	MH251953	–	MH251969
	COUFAL0145	*N. cochenillifera*, Brazil	MH251954	–	MH251970
	COUFAL0146	*N. cochenillifera*, Brazil	MH251955	–	MH251971
*N. novaehollandiae*	CBS 122070	*Grevillea agrifolia*, Australia	–	–	MT592759
	CBS 122072	*Adansonia gregorii*, Australia	–	–	MT592761
	CBS 122610	*Acacia synchronicia*, Australia	–	–	MT592762
	WAC13275	*Mangifera indica*, Australia	GU172400	–	–
	WAC13303	*M. indica,* Australia	GU172398	–	–
*N. orchidacearum*	CMU287	*Cattleya* sp., Thailand	KY933091	KY933092	–
	MFLUCC 12-0533	Orchidaceae, Thailand	KU179865	KU179864	–
*Phyllostica citricarpa*	CBS 102374	*Citrus aurantium*, Brazil	FJ538313	DQ377877	–
*Phyllostica parthenocissi*	CBS 111645	*Parthenocissus quinquefolia*, USA	EU683672	–	–

* Bold letters indicate samples from this study.

## Data Availability

The DNA sequences data obtained in this study have been deposited in GenBank with accession numbers for ITS (LC660640), LSU (LC660641), and *tub* (LC660642).
